# Femoral access site closure without prior femoral angiography: A retrospective analysis

**DOI:** 10.1007/s00508-018-1314-3

**Published:** 2018-01-24

**Authors:** Christoph Brenner, Julian Margreitter, Alexandra Gratl, Josef Klocker, Rudolf Kirchmair, Peter Marschang, Guy Friedrich, Bernhard Metzler, Nicolas Moes

**Affiliations:** 1 Department of Internal Medicine III, Cardiology and Angiology, Medical University of Innsbruck, Anichstr. 35, 6020 Innsbruck, Austria; 2 Department of Vascular Surgery, Medical University of Innsbruck, Innsbruck, Austria

**Keywords:** Radiation protection, Cost-benefit analysis, Vascular system injuries, Cardiac catheters, Safety

## Abstract

**Aims and background:**

Although guideline recommendations have shifted towards a transradial route, femoral puncture is still an established vascular access, especially for complex coronary interventions. The FemoSeal™ vascular closure device (FVCD) helps to reduce femoral compression time and access site complications after removal of the catheter sheath. To ensure safe use, an angiography of the femoral artery prior to FVCD deployment is recommended by the manufacturer. We postulate that omitting this angiography does not relevantly increase the risk for vascular complications.

**Methods and results:**

In this retrospective analysis of an all-comers population (*n* = 1923) including patients receiving a percutaneous coronary intervention (PCI), we could show that combined vascular complication rates without femoral angiography were low (primary endpoint 4.6%) and comparable to a randomized clinical trial that did perform angiography of the vascular access site in a cohort of patients receiving diagnostic coronary angiography only. In addition to this analysis, we could demonstrate that patients with an acute coronary syndrome, receiving periprocedural anticoagulation or anti-platelet therapy had an increased risk for the formation of arterial pseudoaneurysms; however, we did not observe any ischemic vascular event after FVCD deployment.

**Conclusion:**

Closure of the femoral access site after coronary angiography using the FVCD can be safely performed without femoral angiography; however, due to an increased risk for the formation of pseudoaneurysms we recommend the transradial access in situations with increased bleeding risk.

## Introduction

Despite increasing rates of radial access for percutaneous coronary angiography, the femoral vascular access site is still frequently used, especially for complex coronary interventions with use of larger guiding catheters [1]. In previous analyses total vascular complication rates (bleeding and other vascular complications) after femoral puncture for percutaneous interventions ranged from 2% to 7.9% after manual compression for closure of the vascular entry site [2, 3].

The FemoSeal™ vascular closure device (FVCD) has been introduced to reduce femoral compression time and access site complications after removal of the catheter sheath [3, 4]. Previous clinical trials have suggested that the use of VCD may be associated with a slight increased risk for limb ischemia. Vascular stenoses or arterial embolization induced by the FVCD can lead to a critical reduction of blood flow with subsequent need for vascular surgery. Therefore, an angiography of the ipsilateral common femoral artery is recommended by the manufacturer before deployment of the FVCD [5]. This helps to ensure that the femoral artery (1) has a sufficient lumen diameter of ≥5 mm (2), no relevant stenosis, atherosclerotic plaques or vascular abnormalities at the puncture site and that (3) the arterial puncture is not located at or distal to the common femoral artery bifurcation; however, in high-volume centers frequently using the transfemoral approach for coronary angiography, the final femoral angiogram is time-consuming, can lead to a relevant additional radiation exposure for physicians and an increased consumption of the cost-intensive and nephrotoxic contrast agent.

In our center, we generally deploy the FVCD without a prior femoral angiogram. In this study we quantified vascular access site complications resulting from femoral sheath insertions for coronary angiography in 1923 consecutive patients.

## Material and methods

### Patient population

All patients (*n* = 1923) who underwent coronary angiography (CAG) from a femoral access site from July to December 2014 in our hospital received the FVCD after the examination had been completed. At that time, the transfemoral route was still the preferred way of vascular access in our center. We recorded all vascular events requiring further manual, interventional or surgical treatment from the time point of CAG until 2 months thereafter. Our hospital is the only center in the region of North Tyrol (approximately 10,600 km^2^ , 620,000 inhabitants) that offers invasive coronary diagnostics and thus can warrant a reliable clinical follow-up also in retrospective clinical trials. After deployment of the FVCD, all patients received a compression bandage and had to lie in bed with an upper body elevation of not more than 30 ° for 6 h.

### FVCD device

The VCD used in our trial was the FemoSeal™ system manufactured by St. Jude Medical (Plymouth, MN, USA). The FemoSeal™ system can close an arterial puncture of the femoral artery using two resorbable polymer discs connected by a resorbable multifilament. The discs cover the arteriotomy and thus achieve mechanical hemostasis. A detailed description is available from the manufacturer and has previously been published [6].

### Study design

Our investigation was performed as an investigator-initiated retrospective single-center trial.

### Endpoints

The primary endpoint was the incidence of complications at the femoral access site after FVCD administration, i. e. any bleeding with need of manual compression or FemoStop™ administration, pseudoaneurysm (PA), arteriovenous fistula (AVF), local infection or ipsilateral leg ischemia. Bleeding with need for additional compression was defined as any ongoing clinical signs of hemorrhage (palpable subcutaneous swelling/hematoma or bleeding from the puncture site) after deployment of the FVCD. Arterial PA were treated if the diameter exceeded 1 cm. Ultrasound-guided thrombin injection served as first line treatment for arterial PAs. Patients with unfavorable anatomy (as assessed by an angiologist) or unsuccessful thrombin injection were referred to vascular surgery. Arteriovenous fistulas were regarded as relevant if a typically altered blood flow profile was detected in the ipsilateral common femoral vein together with an increase of blood flow of at least 200 ml/min as compared to the contralateral vein. Local infection was defined as any purulent inflammation at the puncture site. Ipsilateral leg ischemia was defined as acute leg ischemia after FCVD deployment or symptoms of a peripheral arterial occlusive disease stadium 2b or higher.

Secondary endpoints were the occurrence of vascular complications in patient subgroups with pre-existing peripheral artery disease (PAD), acute coronary syndrome on admission or the readministration of the FVCD within 90 days at the same puncture site. Other subgroups for secondary analyses received P2Y12 blockers, heparin, bivalirudin or glycoprotein (GP) IIb/IIIa inhibitors.

### Outcome assessment

Outcomes were closely recorded when occurring during and after the patients’ index procedure or when patients were readmitted due to access site complications. For this purpose electronic patient files comprising the period from July 2014 to February 2015 were investigated by two independent investigators.

### Interventional operators and method of access

The team of operators consisted of 14 cardiologists with an approximate mean interventional experience of 11.3 years. All operators used the landmark technique for determining the optimal femoral access point.

### Statistical analysis

We used Graph Pad Prism version 7.01 (GraphPad Software, Inc., La Jolla, CA, USA) for statistical calculations. Fisher’s exact test was used to investigate the impact of pre-existing conditions on the occurrence of vascular complications. A value of *p* < 0.05 was considered statistically significant.

### Approval of the ethics committee

This retrospective analysis has been approved by the Ethics Committee of the Medical University of Innsbruck, Austria (AN2015-0244 354/4.13). Due to the retrospective study design, obtaining a written informed consent from the patients was not regarded necessary by the ethics committee. The trial has been performed in accordance with the ethical standards laid down in the 1964 Declaration of Helsinki.

## Results

### Baseline clinical and demographic patient characteristics

A total of 1923 patients were investigated during this study. Table 1 summarizes the clinical and baseline characteristics. The mean age of the study patients was 67 years, 31.7% were female. Presence of cardiovascular risk factors was common in our population: arterial hypertension (81.3%), hypercholesterolemia (63.8%), diabetes mellitus (18.1%), positive family history for premature cardiovascular diseases (30.2%) and chronic renal failure (28.6%). More than 20% of our study patients were admitted for CAG due to an acute coronary syndrome. Regarding periprocedural anticoagulation and antiplatelet therapy (AA), 1501 (78.3%) of the patients received acetylsalicylic acid, 914 (47.8%) were under treatment with P2Y12 receptor blockers, 261 (13.6%) were orally anticoagulated and 909 (47.5%) received other medication increasing the risk of bleeding at the time of CAG (Table 2). In total, 1325 patients (68.9%) received a 6 French (F) femoral catheter sheath, 598 patients (31.1%) received a 7 F sheath. We used 7 F sheaths for percutaneous coronary intervention (PCI) of more complex lesions (e. g. bifurcations) and for the treatment of patients presenting with an acute coronary syndrome. In general, we already used 7 F sheaths for coronary diagnostics in patients presenting with acute coronary syndrome due to the associated high probability for the need of a subsequent PCI to avoid a time-consuming sheath exchange (from 6 to 7 F) in case of a complex coronary lesion.

**Table 1:** Baseline clinical and demographic patient characteristics

	Number of patients (*n* = 1923)^a^	% of study population
Age (years), median (IQR)	67.00 (58.00–74.00)	–
Female sex	609	31.7
Arterial hypertension	1563	81.3
Hypercholesterolemia	1227	63.8
Diabetes mellitus	349	18.1
– On insulin treatment	118	6.1
Positive family history for premature coronary artery disease	580	30.2
Prior percutaneous coronary intervention	703	36.6
Prior coronary artery bypass graft	85	4.4
Body mass index, median (IQR)	26.23 (24.11–29.06)	–
Renal failure (GFR < 60 ml/min)	550	28.6
– On dialysis treatment	39	2.0
Peripheral artery disease (*n* = 1912)^b^ (stage I, II, III, IV)	106 (63, 35, 2, 6)	5.5 (3.3, 1.8, 0.1, 0.3)
Platelet count (10^9^/L), median (IQR)	207 (175–245)	–
ACS on admission (*n* = 1916)^b^	388	20.2
Arterial blood pressure during coronary angiography (mm Hg), median (IQR)	147 (131–165) systolic	–
	74 (65–81) diastolic	

*IQR* interquartile range, *GFR* glomerular filtration rate, *ACS* acute coronary syndrome

^a^unless otherwise indicated

^b^data available for the specified number of patients

**Table 2:** Antithrombotic medication and anticoagulation before administration of femoral closure device

	Number of patients	% of study population
Acetylsalicylic acid (*n* = 1916)^1^	1501	78.3
P2Y12 receptor blocker (*n* = 1915)^a^	914	47.8
– Clopidogrel	575	30.0
– Prasugrel	147	7.7
– Ticagrelor	192	10.0
Oral anticoagulation (*n* = 1913)^a^	261	13.6
– Vitamin K antagonists	86	4.5
– Rivaroxaban	127	6.6
– Dabigatran	15	0.8
– Apixaban	33	1.7
Others (*n* = 1915)^a^	909	47.5
– Tirofiban	14	0.7
– Abciximab	50	2.6
– Heparin	770	40.2
– Bivalirudin	75	3.9

^a^data available for the specified number of patients

Demographics as well as presence of cardiovascular risk factors were comparable to study populations investigated in previous, randomized clinical trials [3, 6].

### Clinical outcome

The primary endpoint occurred in 89 patients (4.6%). Access site-related bleeding after deployment of the FVCD could be controlled with manual compression in 30 patients (1.6%) or use of the FemoStop™ system in 21 patients (1.1%). Other access site-related complications were documented in 44 patients (2.3%, PA) and 1 patient (0.05%, AVF with need for surgical treatment). Local infections occurred in none of the patients. Importantly, we did not see a case of ipsilateral lower limb ischemia (Table 3).

**Table 3:** Outcomes within 56 days after administration of femoral closure device

	Number of patients (*n* = 1923)	% of study population
Access site-related bleeding with need of manual compression	30	1.6
Access site-related bleeding with need of FemoStop™ administration	21	1.1
Pseudoaneurysm	44	2.3
– Manual compression	25	1.3
– Fibrin coagulation	7	0.4
– Surgical treatment	12	0.6
Arteriovenous fistula with surgical treatment	1	0.05
Local infection	0	–
Ipsilateral leg ischemia	0	–
Any bleeding with need of manual compression or FemoStop™ administration, pseudoaneurysm, arteriovenous fistula, local infection or ipsilateral leg ischemia	89	4.6

In our subgroup analyses the presence of an acute coronary syndrome on admission increased the risk for developing postinvasive PA (4.4% vs. 1.8%, *p* < 0.01). The presence of a PAD did not significantly affect the risk for vascular access site complications (*p* = 0.20). Also, the readministration of the FVCD within 90 days at the same femoral artery, which occurred in 117 patients, did not increase the risk for vascular complications (Table 4).

**Table 4:** Subgroup analysis of vascular preconditions

	Number of patients with pre-existing condition	% of population	Number of patients without pre-existing condition	% of study population	*p*-value
Peripheral artery disease (*n* = 1912)^a^	106	–	1806	–	–
– Access site-related bleeding with need for compression (manual or FemoStop™)	5	4.7	46	2.5	0.20
– Pseudoaneurysm	0	0	44	2.4	0.17
Acute coronary syndrome on admission (*n* = 1916)^a^	388	–	1528	–	–
– Access site-related bleeding with need for compression (manual or FemoStop™)	11	2.8	40	2.6	0.86
– Pseudoaneurysm	17	4.4	27	1.8	<0.01
Readministration of the femoral closure device within 90 days at same puncture site (*n* = 1920)^a^	117	–	1803	–	–
– Access site-related bleeding with need for compression (manual or FemoStop™)	1	0.9	50	2.7	0.37
– Pseudoaneurysm	2	1.7	42	2.3	1.00

^a^data available for the specified number of patients

Pharmacological pretreatment of the patients with P2Y12 blockers (3.5% vs. 1.2%, *p* < 0.01) or anticoagulants (heparin or bivalirudin, 3.3% vs. 1.5%, *p* < 0.01) increased the risk for developing PA but did not compromise the primary hemostasis mediated by the FVCD (Table 5). The need for additional compression after deployment of the FVCD (manual compression or using the FemoStop™ system) was neither increased in patients with increased vascular risk (PAD, ACS, readministration of FVCD, Table 4) nor in subjects with a pharmacologically increased risk for bleeding (antiplatelet therapy, anticoagulation). In further detailed analyses we could detect a numerically higher number of PAs in patients with an ACS in the cohort of anticoagulated patients (4.9% vs. 2.4%, *p* = 0.07). We could also observe a numerically higher number of PAs in patients receiving anticoagulants in the cohort of non-ACS patients (2.5% vs. 1.3%, *p* = 0.11). Both observations were not statistically significant (Table 5).

**Table 5:** Subgroup analyses of pharmacological preconditions

	Number of patients with pre-existing condition	% of population	Number of patients without pre-existing condition	% of study population	*p*-value
Administration of P2Y12 receptor blockers (*n* = 1915)^1^	914	–	1001	–	–
– Access site-related bleeding with need for compression (manual or FemoStop™)	21	2.3	30	3.0	0.39
– Pseudoaneurysm	32	3.5	12	1.2	<0.01
Administration of heparin or bivalirudin (*n* = 1915)^a^	845	–	1070	–	–
– Access site-related bleeding with need for compression (manual or FemoStop™)	22	2.6	29	2.7	1.00
– Pseudoaneurysm	28	3.3	16	1.5	<0.01
Patients with ACS in the cohort of patients receiving heparin or bivalirudin (*n* = 845)	304	–	541	–	–
– Access site-related bleeding with need for compression (manual or FemoStop™)	7	2.3	14	2.6	1.00
– Pseudoaneurysm	15	4.9	13	2.4	0.07
Administration of heparin or bivalirudin in the cohort of patients without ACS (*n* = 1528)	552	–	976	–	–
– Access site-related bleeding with need for compression (manual or FemoStop™)	14	2.5	26	2.6	1.00
– Pseudoaneurysm	14	2.5	13	1.3	0.11
Administration of GP IIb/IIIa inhibitors (*n* = 1915)^a^	64	–	1851	–	–
– Access site-related bleeding with need for compression (manual or FemoStop™)	1	1.6	50	2.7	1.00
– Pseudoaneurysm	4	6.2	40	2.2	0.06
Administration of any P2Y12 blocker, heparin/bivalirudin or GP IIb/IIIa inhibitor (*n* = 1911)^a^	1063	–	848	–	–
– Access site-related bleeding with need for compression (manual or FemoStop™)	29	2.7	22	2.6	0.89
– Pseudoaneurysm	33	3.1	11	1.3	<0.01
Administration of any P2Y12 blocker (prasugrel/ticagrelor vs. clopidogrel) (*n* = 914)	339 (prasugrel/ticagrelor)	–	575 (Clopidogrel)	–	–
– Access site-related bleeding with need for compression (manual or FemoStop™)	8	2.4	13	2.3	1.0
– Pseudoaneurysm	6	1.8	22	3.8	0.11

^a^data available for the specified number of patients

*ACS* acute coronary syndrome, *GP* glycoprotein

## Discussion

In this retrospective analysis of patients receiving the FVCD without a prior femoral angiography, we could demonstrate that local vascular complications, i. e. any bleeding with additional need for manual compression or FemoStop™ administration, PA, AVF, local infection or ipsilateral leg ischemia, occurred in 4.6% of our patients. The vascular complication rate was largely comparable to the number of complications reported in a large-scale randomized clinical trial (ISAR-CLOSURE) which, in contrast to our trial, performed femoral angiography prior to FVCD deployment as recommended by the manufacturer [3]. Due to the different study designs, endpoint definitions could not be completely matched in both trials; however, we were able to demonstrate that using the FVCD without prior angiography is relatively safe. This may help to reduce radiation exposure for physicians and to diminish use of contrast agent. Furthermore, major vascular complication rates were comparably low. Our study included all clinically relevant endpoints which make the study results of both trials largely comparable.

### Pseudoaneurysms

Compared to the trial by Schulz-Schupke et al. we observed a slightly higher number of PA in our study population [3]. This may be based on the fact that our all-comers population partially presented in an acute clinical setting (20.2% of patients). In contrast to patients presenting with stable angina pectoris, ACS treatment comprised the predominant use of 7 F catheter sheaths as well as administration of anticoagulants and antiplatelet therapy. Both the presence of ACS as well as administration of anticoagulants led to an increase in PA formation. Administration of oral P2Y12 inhibitors also increased the rate of femoral pseudoaneurysms. The PAs occurred in more than 3% of our patients that were treated with any P2Y12 blocker, heparin/bivalirudin or a GP IIb/IIIa inhibitor. This reflects the fact that AA can lead to a persistent minor hemorrhage at the femoral puncture site after use of a VCD [1]. Patients not treated with AA therapy in our population as well as in the ISAR-CLOSURE trial showed a significantly lower PA rate of 1.3% and 1.8%, respectively [3].

We saw a numerical increase of PA in the setting of ACS when only patients receiving anticoagulants were observed. Likewise, we could detect a trend towards increased numbers of PA in patients receiving anticoagulants when only patients without ACS were observed. Although not significant, both observations support the hypothesis that the presence of ACS as well as administration of anticoagulants increase the risk of PA formation independent from each other. While heparin administration might increase the risk of bleeding at the site of sheath introduction, we can speculate that the setting of ACS might lead to an increased number of PAs due to inaccurate arterial puncture or restless patients in emergency situations. Due to a huge overlap in patient subgroups receiving 7 F sheaths, presenting with ACS or receiving anticoagulants, we were not able to identify the use of 7 F sheaths as an independent risk factor for the development of arterial PA; however, although the deployment of the FVCD is safe to seal 7 F puncture sites [7], we cannot exclude that the larger sheath diameter did have an impact on PA formation.

### External compression

The AA treatment did not adversely affect primary hemostasis, i. e. the need for additional external compression (manual compression or using the FemoStop™ device) immediately after deployment of the FVCD. Corresponding to our results, another randomized clinical trial, the CLOSE-UP study, showed comparably low numbers of vascular complications after FVCD deployment without prior femoral angiography [6]. Thus, regarding the integrity of the femoral arterial wall, we can summarize that deployment of the FVCD appears to be safe without prior angiography of the punctured artery.

### Ischemic events

Beside hemorrhagic complications, the use of VCDs bears the risk for induction of vascular stenoses or occlusions as well as embolization of the closure device. We did not see a single ischemic vascular event that had to be treated surgically or interventionally after deployment of the FVCD. These results are in line with both the CLOSE-UP and the ISAR-CLOSURE trials that did not report any ischemic events in the lower limb after FVCD deployment, as well [3, 6].

### Limitations

The major limitation of our trial is the retrospective analysis of clinical data routinely recorded from the patients referred to coronary angiography and angioplasty in our hospital. We therefore did not quantify hematomas or vascular complications that did not lead to interventional or surgical treatment. The study design also comprises the fact that we were not able to compare our data to a fully matched control population. Although our baseline and demographic patient characteristics well reflect the typical western patients suffering from coronary artery disease, certain differences to comparable populations from randomized trials still exist. We notably included patients suffering from ACS who were not represented in the two available large randomized clinical trials [3, 6]; however, non-ACS patients in our population showed the same rates of PA as compared to the ISAR-CLOSURE trial (1.8%). A final consideration of risks and benefits of omitting the femoral angiography prior to FVCD deployment can only be determined in a future randomized clinical trial.

## Conclusion

Large-scale randomized trials have only investigated the use of the FemoSeal™ device in patients receiving diagnostic coronary angiography using a 6 F sheath. Patients presenting with ACS, undergoing PCI or receiving anticoagulants have been excluded from all of these studies [3, 6].

In this retrospective analysis, we could now show that omitting femoral angiography prior to the deployment of the FemoSeal™ system appears to be safe in an all-comers population undergoing diagnostic coronary angiography and PCI with stable angina pectoris or acute coronary syndrome using catheter sheaths up to a size of 7 F. The overall complication rates were comparable to those seen in the studies mentioned above.

While FemoSeal™ reimplantation at the same site during a time frame of 90 days as well as the presence of a PAD did not affect the rate of vascular complications, we could see that patients undergoing PCI for the treatment of an ACS or stable angina showed increased numbers of arterial PA. This supports the current guideline recommendations for a preferred use of the radial access route, especially in situations with an increased risk of bleeding [8–10].

## Abbreviations

AA: Periprocedural anticoagulation and anti-platelet therapy
ACS: Acute coronary syndrome
CAG: Coronary angiography
F: French (0.33 millimeter)
FVCD: FemoSeal™ vascular closure device, manufactured by St. Jude Medical
GFR: Glomerular filtration rate
IQR: Interquartile range
PA: Pseudoaneurysm
PAD: Peripheral artery disease
VCD: Vascular closure device
